# Improving the utility and sustainability of novel health technology to improve clinical outcomes for patients: an East Staffordshire experience of screening for atrial fibrillation with the AliveCor KardiaMobile

**DOI:** 10.3399/BJGPO.2020.0169

**Published:** 2021-02-24

**Authors:** Savio Mathew, Ruth Chambers

**Affiliations:** 1 Clinical Teaching Fellow, University of Leicester, Leicester, UK; 2 Clinical Lead for Technology Enabled Care Service (TECS) Programme, Staffordshire Sustainability and Transformation Partnership’s Digital Workstream, Stoke-on-Trent, UK

**Keywords:** Screening, Cardiovascular disease, Diagnosis, Atrial fibrillation, Surveys and questionnaires

## Background

Atrial fibrillation (AF) is the most common sustained arrythmia in the world.^1^ With a two-fold increased risk of mortality, a near five-fold increased risk of stroke, and a two- to three-fold increased risk of heart failure, the rising prevalence of AF is a major public health burden.^2^ Patients typically present with palpitations, chest pain, or breathlessness; however, up to a third of patients are asymptomatic. Identifying this subgroup and commencing them on anticoagulation therapy if justified is therefore a major obstacle and necessity in the secondary prevention of AF.^3,4^

The use of novel health technology in this regard is an option with much potential. Across East Staffordshire Clinical Commissioning Group (CCG), we embraced this potential and piloted an initiative to improve identification of patients with AF by distributing single-lead electrocardiogram (ECG) devices to 18 general practices between October 2019 and March 2020, following an initial workshop as part of a CCG Local Incentive Scheme. Screening data, as well as clinical feedback in the form of a questionnaire using the online platform Google forms, were then collected from the practices. Further, quality and outcomes framework (QOF) data from NHS Digital and Public Heath England were analysed for trends in the CCG and region.^5,6^

We present five insights from our experiences and highlight how single lead ECG devices were used to improve clinical outcomes for patients across East Staffordshire.

### Insight 1: Choose a suitable product/novel technology

Identifying a suitable solution for the need being addressed is vital to the successful implementation of novel technology. In the diagnosis of AF, 12-lead ECG is the gold-standard choice of investigation.^7^ However, these tend to be time-consuming during busy clinics, and are dependent on the accuracy of the recording and its subsequent interpretation by a trained clinician. To negate this, we deployed AliveCor KardiaMobile, a portal single-lead ECG device that requires patients to place their fingers on the device for 30 seconds. Recordings are sent wirelessly to compatible smartphone devices and can be viewed on the accompanying Kardia app. The device was chosen for its ease of use and reported range of sensitivity between 77.0% and 96.6% and specificity between 76.0% and 99.1% in the detection of cardiac arrhythmia.^8^

### Insight 2: Engage key stakeholders

Early identification and engagement of key stakeholders facilitates early adoption and implementation of health technology.^9^ Key stakeholders we identified included clinicians, practice managers, CCG leads, and the primary care network clinical director. Early engagement facilitated a shared vision and objective among all parties involved with the CCG Chair presenting at the initial workshop. Not only did this facilitate greater uptake and implementation of this technology, it advanced our efforts to gather feedback to drive the project forward in the latter stages with the support of the commissioned data facilitation from engaged practices.

### Insight 3: Engage users and reduce barriers

Engaging target users and addressing barriers in the uptake and utility of novel technology is another key determinant. In this regard, we invited clinicians and practice staff across East Staffordshire to attend an educational workshop, where talks on best practices in detecting and managing AF, and training on how to use the AliveCor KardiaMobile, were delivered prior to distributing AliveCor devices. Targeted education in this manner reduced barriers around the use of devices and improved utility of the devices, as it required a level of engagement from users. It further raised awareness of the need for early diagnosis of AF and management strategies among all participants.

### Insight 4: Be opportunistic

Opportunistic use of technology was another key insight from our experiences. Feedback from clinicians showed differences in the way the AliveCor devices were used. Most notable among them was the indiscriminate use of the devices during flu vaccination clinics. This initiative detected several asymptomatic patients who were subsequently diagnosed with AF. Other examples included the use of these devices during home visits and after the detection of an irregular pulse on physical examination.

### Insight 5: Gather feedback

Gathering of feedback and acting on that is another key driver of success. In this regard, we sent out a brief questionnaire using Google forms to stakeholders, to gain further insights into the utility of the devices in the community. Amid the ongoing COVID-19 global pandemic, we further probed stakeholders as to how the pandemic changed utility of the devices. As expected, there was great heterogeneity in the way the devices were used, and some barriers were identified. Nevertheless, most of stakeholders found the devices useful and would recommend them to a colleague.

## Results

Of the 18 practices within East Staffordshire CCG, 16 practices actively participated in this initiative. Eighty AliveCor devices were given out to these practices. In total, 3772 patients (not previously diagnosed with AF) were screened for AF with AliveCor devices in the 6-month period, of whom the majority were attending flu vaccination clinics and were aged ≥65 years. Of these, 258 patients were indicated to have probable AF by the AliveCor devices. A 12-lead ECG confirmed AF for 49 of these patients (positive predictive value = 19%). Forty were subsequently commenced on anticoagulation therapy, with 61% of the newly diagnosed AF patients being managed in practice.

We received 18 responses to our e-survey request for feedback from practice teams. Responses were received from GPs, practice managers, practice nurses, and health care assistants. Ninety-four per cent of responders found the device useful and would recommend it to another colleague. The one responder who found the device redundant commented that if they noticed an irregular pulse manually, they would always do a 12-lead ECG, and therefore did not find the device useful. In response to changes in practice with the current COVID-19 pandemic, the vast majority found the devices less useful due to reduced face-to-face consultations, time constraints, and infection control measures.

In comparison to the national average, the AF prevalence in East Staffordshire CCG has continued to increase annually and is marginally greater in 2019/20, at 2.21 versus 2.05.^5^ Further, among patients with AF with a CHADVASC^10^ score ≥2, the percentage of patients treated with anticoagulation drug therapy was found to be markedly higher than the national average at 94.47 vs 91.79.

## Conclusion

The results of this study show a positive impact on the diagnosis and management of AF using AliveCor KardiaMobile devices in East Staffordshire. Not only have they contributed to improved patient outcomes, with 49 new diagnoses of AF in otherwise asymptomatic patients, but they also have widespread acceptance and are valued by the clinicians utilising them. These findings are in keeping with other studies that have reported similar value in utilising AliveCor KardiaMobile in the detection of AF, and with European Society of Cardiology guidelines that recommend opportunistic screening for AF in patients ≥65 years of age.^10,11^

Through this technology, we hope to continue improving the diagnosis of AF and associated best practice clinical management, and so reduce the personal, emotional, and financial burden of its complications (such as an avoidable stroke). We have demonstrated here that novel health technology can be used in real life practice and should be considered for addressing challenges in the community. In the implementation of such solutions, from our experiences, a multidimensional approach to the clinical implementation of such strategy is often the best means to achieve sustainability and greater utility.
